# *Xenopus gpx3* Mediates Posterior Development by Regulating Cell Death during Embryogenesis

**DOI:** 10.3390/antiox9121265

**Published:** 2020-12-12

**Authors:** Hongchan Lee, Tayaba Ismail, Youni Kim, Shinhyeok Chae, Hong-Yeoul Ryu, Dong-Seok Lee, Taeg Kyu Kwon, Tae Joo Park, Taejoon Kwon, Hyun-Shik Lee

**Affiliations:** 1 KNU-Center for Nonlinear Dynamics, CMRI, BK21 Plus KNU Creative BioResearch Group, School of Life Sciences, College of Natural Sciences, Kyungpook National University, Daegu 41566, Korea; leehongchan@hanmail.net (H.L.); tayabaismail@gmail.com (T.I.); poloqq@hanmail.net (Y.K.); rhr4757@knu.ac.kr (H.-Y.R.); lee1@knu.ac.kr (D.-S.L.); 2 Department of Biomedical Engineering, Ulsan National Institute of Science and Technology (UNIST), College of Information-Bio Convergence, Ulsan 44919, Korea; szszorol@unist.ac.kr; 3 Department of Immunology, School of Medicine, Keimyung University, Daegu 42601, Korea; kwontk@dsmc.or.kr; 4 Department of Biological Sciences, Ulsan National Institute of Science and Technology (UNIST), College of Information-Bio Convergence, Ulsan 44919, Korea; parktj@unist.ac.kr

**Keywords:** gpx3, tailbud, apoptosis, posterior development, embryogenesis

## Abstract

Glutathione peroxidase 3 (GPx3) belongs to the glutathione peroxidase family of selenoproteins and is a key antioxidant enzyme in multicellular organisms against oxidative damage. Downregulation of GPx3 affects tumor progression and metastasis and is associated with liver and heart disease. However, the physiological significance of GPx3 in vertebrate embryonic development remains poorly understood. The current study aimed to investigate the functional roles of *gpx3* during embryogenesis. To this end, we determined *gpx3*’s spatiotemporal expression using *Xenopus laevis* as a model organism. Using reverse transcription polymerase chain reaction (RT-PCR), we demonstrated the zygotic nature of this gene. Interestingly, the expression of *gpx3* enhanced during the tailbud stage of development, and whole mount in situ hybridization (WISH) analysis revealed *gpx3* localization in prospective tail region of developing embryo. *gpx3* knockdown using antisense morpholino oligonucleotides (MOs) resulted in short post-anal tails, and these malformed tails were significantly rescued by glutathione peroxidase mimic ebselen. The gene expression analysis indicated that *gpx3* knockdown significantly altered the expression of genes associated with Wnt, Notch, and bone morphogenetic protein (BMP) signaling pathways involved in tailbud development. Moreover, RNA sequencing identified that *gpx3* plays a role in regulation of cell death in the developing embryo. Terminal deoxynucleotidyl transferase dUTP nick end labeling (TUNEL) and phospho-histone 3 (PH3) staining confirmed the association of *gpx3* knockdown with increased cell death and decreased cell proliferation in tail region of developing embryos, establishing the involvement of *gpx3* in tailbud development by regulating the cell death. Furthermore, these findings are inter-related with increased reactive oxygen species (ROS) levels in *gpx3* knockdown embryos, as measured by using a redox-sensitive fluorescent probe HyPer. Taken together, our results suggest that *gpx3* plays a critical role in posterior embryonic development by regulating cell death and proliferation during vertebrate embryogenesis.

## 1. Introduction

Reactive oxygen species (ROS) are highly reactive chemical species that contain oxygen in the form of reactive superoxide anions, hydrogen peroxides, or hydroxyl radicals [1]. In recent years, ROS have been recognized as important signaling molecules that play a role in early embryonic development and regeneration [2]. Maintaining high levels of hydrogen peroxide is required for Wnt/β-catenin signaling and fibroblast growth factor (FGF) signaling activation during *Xenopus* tail regeneration [3]. An ROS burst is observed after fertilization in sea-urchins and *Xenopus* [4]. ROS play essential roles during egg cleavage and mesoderm formation during vertebrate embryogenesis [5]. However, excessive ROS damage can affect various cells and biological processes [1,6]. Therefore, ROS homeostasis is required for steady-state biological and cellular processing [1,6,7].

Cells use different enzymatic and non-enzymatic antioxidants to maintain ROS homeostasis [6,7]. Glutathione peroxidase (GPx) is a multiple isozyme group that is involved in converting hydrogen peroxide (H_2_O_2_) or organic hydrogen peroxide to water using reduced glutathione as an electron donor [8]. Some GPxs have selenium-dependent glutathione peroxidase activity [9]. In mammals, four selenium dependent GPxs are broadly distributed in different mammalian tissues: GPx1, GPx2, Gpx3, and GPx4, [10].

Glutathione peroxidase 3 (GPx3), also known as plasma GPx, is present in various organs, including kidney, lung, heart, muscles, epididymis, vas deferens, placenta, and seminal vesicles [11,12,13]. GPx3 forms a homotetramer and is a secreted protein that is also present in the cytoplasm [14]. GPx3 plays a crucial role in ROS detoxification. Studies have shown that GPx3 silencing promotes tumor metastasis in human gastric and thyroid cancer [15]. Recent reports suggest that GPx3 ablation enhances tumor inflammation, injury, proliferation, and DNA damage in mice subjected to inflammatory carcinogenesis and chronic colitis [16]. Hyper-methylation-mediated downregulation of GPx3 was also observed in breast, ovarian, gastric, esophagus, and prostate cancer [17,18,19,20,21]. In addition to its anticancer role, high GPx3 levels alleviate cardiovascular disease-associated mortality [22]. GPx3 downregulation enhances liver aging, and an inadequate GPx3 level is a significant factor in human heart disease [23,24]. However, GPx3’s role during early embryonic development remains elusive.

In this study, we investigated *gpx3* developmental functions during *Xenopus* embryogenesis. Analyzing *gpx3*’s spatiotemporal expression pattern revealed that it is expressed in the tailbud region. *gpx3* knockdown resulted in a shortened post-anal tail and increased ROS levels. Whole mount in situ hybridization (WISH) analysis indicated that *gpx3* is not involved in the tailbud structure. However, *gpx3* depletion induced cell death and decreased cell proliferation in the tailbud region. These findings suggest that *gpx3* plays a significant role in the tailbud extension during *Xenopus* embryonic development.

## 2. Materials and Methods

### 2.1. Xenopus Growth Conditions and In Vitro Fertilization

Adult *X. laevis* were obtained from the Korean *Xenopus* Resource Center for Research and were housed at 18 °C with a 12 h photoperiod in containers recommended by the Ulsan National Institute of Science and Technology Institutional Review Board. To induce ovulation, *Xenopus* females were injected with 1000 IU of human chorionic gonadotropins into the dorsal lymph sac the evening before the experiment. The next morning, females were transferred to a 1x high salt solution for egg harvesting. For fertilization, male *Xenopus* were kept in a 1x benzocaine solution for 5–15 min and were then sacrificed to isolate the testes. The isolated testes were kept in 1x modified Barth’s saline (MBS) at 4 °C. The eggs were washed with 0.1x MBS three times and were then fertilized with a sperm suspension solution derived from the isolated testes. After fertilization, the eggs were de-jellied by swirling them in 2% L-cysteine and were then washed five times with 0.5x MBS. The unfertilized and dead eggs were removed. The fertilized eggs were cultured at 15–18 °C in 0.5x MBS containing 2% Ficoll 400 (GE Healthcare, Little Chalfont, UK) after injections.

### 2.2. Plasmids and mRNA Synthesis

As *gpx3* has a UGA opal codon within the coding sequence, to avoid translation termination and confirm morpholino oligonucleotides (MO) specificity, we cloned a mutant construct that has the selenocysteine changed to cysteine. DNA was isolated from tailbud stage *Xenopus*. *gpx3* primers were designed based on the *gpx3* sequences listed in Xenbase and NCBI. Flag-tagged U73C *gpx3* mRNA was synthesized using PCR, and the plasmid was constructed using the pCS107 vector with ClaI and XhoI restriction sites. 

The HyPer-cyto gene was obtained from pHyPer-cyto (Evrogen, Moscow, Russia) and subcloned into the pCS2+ vector. HyPer-pCS2+ was linearized using the NotI restriction enzyme. SP6 mMessage mMachine kit was used to synthesize mRNA (Ambion, Woodward Street, Austin, TX, USA).

### 2.3. MO Design and Xenopus Embryo Microinjection

A 25-nucleotide *gpx3* MO (Gene Tools) was designed to bind the *gpx3* mRNA translation initiation region. The *Xenopus gpx3* translation-blocking MO sequence was 5′-atgggggtcaaatttagggggctct-3′, and the splicing-blocking MO sequence was 5′-acgcacatacctggcagtcaaaaac-3′. MOs were injected into the embryo’s ventral blastomere at the 4-cell stage, and green fluorescent protein mRNA was co-injected as a lineage tracer. Ebselen, a GPx chemical mimic, was used to perform the rescue experiments.

### 2.4. Whole-Mount In Situ Hybridization

The *gpx3* MO was injected into the *Xenopus* embryos ventral region at the 4-cell stage. The MO-injected embryos were collected at the desired stage and were fixed in fixative MEMFA (4% paraformaldehyde, 0.1 M MOPS buffer (pH 7.4), 1 mM MgSO_4_, 2 Mm ethylene glycol-bis(β-amino ethyl ether)-*N*,*N*,*N*′,*N*′-tetraacetic acid (EGTA) overnight at 4 °C. The embryos were dehydrated before storage in 100% methanol at −20 °C. To prepare the antisense digoxigenin (DIG)-labeling probes, DNA templates were linearized using appropriate restriction enzymes. Probes were synthesized using SP6 or T7 RNA polymerase (Ambion) and were detected using an alkaline phosphatase-labeled antidigoxigenin antibody (1:1000, Roche, Basel, Switzerland) or nitro blue tetrazolium/5-bromo-4-chloro-3indolyl phosphate (NBT/BCIP) [25].

### 2.5. Reverse Transcription Polymerase Chain Reaction

Total RNA was extracted from *Xenopus* embryos using Isol-RNA lysis reagent (5 Prime GmbH, Hilden, Germany). cDNA was synthesized from total RNA of *Xenopus* embryos using the PrimeScript first-strand cDNA synthesis kit (Takara, Kusatsu, Japan). PCR was performed using customized primers, (Table 1) and the products were loaded on 1% agarose gels. Images were captured using WiseCapture I-1000 (Daihan Scientific, Wonju, South Korea).

### 2.6. Transcriptomic Analysis

An RNA sequencing (RNA-seq) library was constructed from total RNA extracted from each sample with polyA enrichment, according to the manufacturer’s instructions (Illumina). To estimate the mRNA abundance, *X. laevis* cDNA sequence reads were mapped from the genome project consortium [26] using bwa (version 0.7.15). EdgeR (version 3.20.7) was used to analyze differentially expressed genes (DEGs). Significantly differentially expressed genes had a >2-fold change with a false discovery rate (FDR) <0.01 in an exact test. Over-represented biological processes in the DEGs were tested using Fisher’s test and PANTHER database (released 20171205). Human orthologous genes were determined based on best hits using the BLASTP search. Raw data for the RNA-seq is available at the NCBI GEO database (PRJEB39362) [27].

### 2.7. Terminal Deoxynucleotidyl Transferase dUTP Nick End Labeling (TUNEL) and Phospho-Histone 3 (PH3) Staining

*Xenopus* embryos were fixed for 24 h in MEMFA at 4 °C and bleached (3% H_2_O_2_, 5% formamide, and 5xsaline sodium citrate (SSC)) after washing with phosphate buffer saline (PBS). The embryos were processed for TUNEL and PH3 staining, as described previously [28].

### 2.8. In Vivo Imaging of ROS

HyPer cyto (HyPer) was used to detect H_2_O_2_ related ROS. HyPer mRNAs (10 ng) were injected into the 1-cell staged embryos. Images were taken using live anesthetized embryos using 1:1000 diluted benzocaine and an Olympus FV1200 confocal microscope. Images were analyzed by ImageJ [Cite Schneider, C. A.; Rasband, W. S. & Eliceiri, K. W. (2012), “NIH Image to ImageJ: 25 years of image analysis”, Nature methods 9(7): 671-675, PMID 22930834 ]. 

### 2.9. Statistical Analysis

WISH and RT-PCR data were analyzed using ImageJ. Results are presented as the mean ± standard error from three independent experiments. Results were interpreted by unpaired t-test (two-tailed) using GraphPad Prism 7 software (GraphPad Software Inc., La Jolla, CA, USA). P-values of less than 0.05 were considered statistically significant. P-values were represented by asterisk (*).

## 3. Results

### 3.1. Gpx3 Spatiotemporal Expression Pattern during Xenopus Embryogenesis

**Figure 1:**
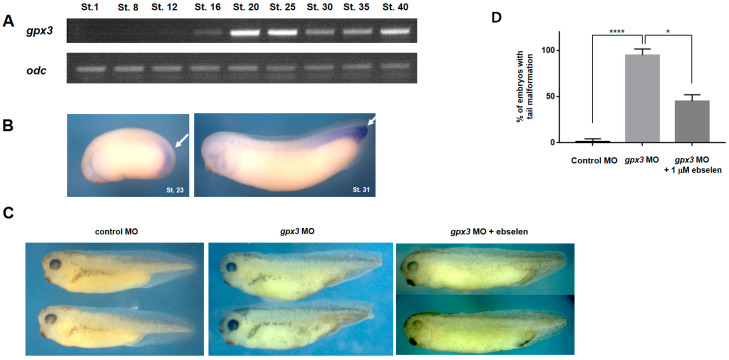
*gpx3* is required for tailbud development during *Xenopus* embryonic development. (**A**) RT-PCR analysis indicated the zygotic *gpx3* gene expression. *gpx3* expression started at the gastrula stage of development (St.12) and continued until later stages (St.40). The highest expression levels were observed at the tailbud stage (St.20-25). Ornithine decarboxylase (*odc*) was used as an internal control. (**B**) Whole mount in situ hybridization (WISH) analysis demonstrated *gpx3* expression at the prospective tailbud region (St.23) and exhibited its localization in the developing embryo tail region (St.31). (**C**) *gpx3* morpholino oligonucleotides (MO) microinjection into the 4-cell stage embryo ventral side resulted in short post-anal tails in *gpx3* morphant embryos compared to control MO-injected embryos. Malformed phenotypes induced by *gpx3* knockdown were effectively rescued by ebselen, a GPx mimic. (**D**) Embryo phenotype quantification revealed that more than 90% of the injected embryos with *gpx3* MO developed short post-anal tails compared to control embryos. The short tail phenotypes were 50% recovered in ebselen-treated embryos. * *p* < 0.05, **** *p* < 0.0001.

To determine the specific *gpx3* roles during *Xenopus* embryonic development, we first analyzed the *gpx3* expression pattern in *Xenopus*. RT-PCR was conducted to assess the temporal *gpx3* expression. RT-PCR indicated that *gpx3* is first expressed at the gastrula stage (Nieuwkoop and Faber; St.12) of *Xenopus* embryos (Figure 1A). The highest expression levels were observed at the tailbud stage (NF. St.20-25). Expression was observed until later development stages, such as the late tailbud stage (St.40; Figure 1A). RT-PCR revealed the zygotic nature of *gpx3*, with the highest expression level at the early tailbud stage in *Xenopus* embryos.

WISH analysis determined the *gpx3* spatial expression during *Xenopus* embryonic development. Our results showed the *gpx3* expression in the developing embryo prospective tailbud region (NF. St.23) and in the tail region of later stage embryos (NF. St. 31; Figure 1B). These results suggest that *gpx3* may be involved in tailbud development during *Xenopus* embryogenesis.

### 3.2. Gpx3 Knockdown Leads to a Reduced Post-Anal Tail during Embryonic Development

To evaluate our hypothesis that *gpx3* is involved in tailbud development, loss of function experiments were performed using antisense MO against *gpx3*. To check the specificity and the efficiency of MO against *gpx3*, we used translational blocking MO and splicing blocking MO. These *gpx3* MOs were microinjected into the ventral region of *Xenopus* embryos at the 4-cell stage to repress *gpx3* expression. Microinjection of the *gpx3* MOs resulted in a shortened post-anal tail in the morphant embryos compared to control embryos (Figure 1C). It was revealed that more than 90% of the embryos injected with the *gpx3* MO exhibited reduced tail development (Figure 1D). 

To further determine the *gpx3* role in tailbud development and to confirm the *gpx3* specificity, we conducted rescue experiments using ebselen, which mimics glutathione peroxidase [29]. Ebselen has free-radical and singlet oxygen quenching properties and catalyzes the hydrogen peroxide reduction similar to glutathione peroxidase at the expense of thiol [29]. The reason for performing rescue experiments using ebselen was the non-availability of *gpx3* specific antibodies in *Xenopus*. Thus, to rescue the *gpx3* MO-induced malformations, we injected *gpx3* MO into the *Xenopus* embryos at the 4-cell stage and transferred them to a 1 μΜ ebselen solution. Approximately 50% of the *gpx3* MO-injected embryos grown in the ebselen media recovered from the malformations that were observed in *gpx3* morphant embryos (Figure 1C,D). Taken together, these findings demonstrate the *gpx3* MO specificity, which is responsible for a shortened post-anal tail in *gpx3* morphant embryos.

### 3.3. Gpx3 Mediates Tailbud Development through Wnt, Notch, and FGF Signaling Pathway Regulation 

**Figure 2:**
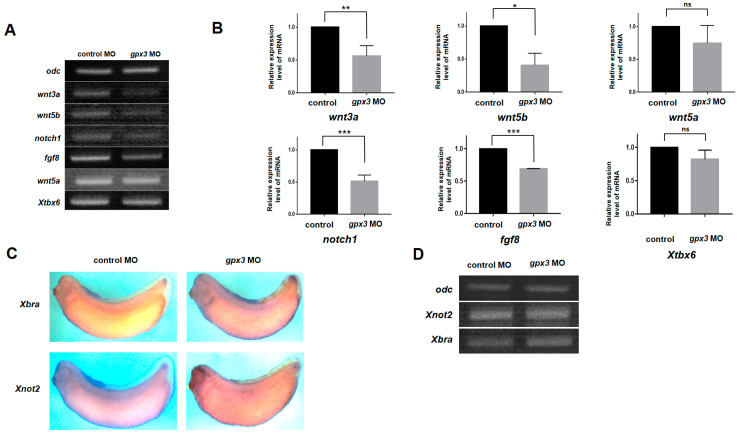
*gpx3* knockdown perturbed Wnt, Notch, and FGF signaling gene expression but did not affect the early tailbud gene expression. (**A**,**B**) *gpx3* MO was injected into the ventral regions of 4-cell stage embryos, and RT-PCR was used to determine *wnt3a*, *wnt5a*, *wnt5b*, *notch1*, *fgf8*, and *Xtbx6* gene expression after removing the anterior part of embryos. *wnt3a*, *wnt5b*, *notch1*, and *fgf8* expressions were significantly reduced in *gpx3* MO-injected embryos. * *p* < 0.05; ** *p* < 0.001; *** *p* < 0.0001; ns, not significant. (**C**) WISH analysis of *gpx3* morphant embryos using *Xbra* and *Xnot2* (chordoneural hinge and posterior wall markers). *gpx3* knockdown did not affect the early tailbud gene expression. (**D**) RT-PCR of *Xbra* and *Xnot2* relative expression revealed no significant differences between the control and *gpx3* MO-injected embryos.

In *Xenopus*, tailbud development is regulated by different signaling pathways. Active Wnt/β-catenin signaling is required for posterior development, and many Wnt signaling genes, such as *wnt3a*, *wnt5*, and *wnt8*, are expressed in the posterior portion of the developing embryos [30,31]. In addition to Wnt, Notch and bone morphogenetic protein (BMP) signaling also play critical roles in tailbud outgrowth [32,33]. FGF proteins are also involved in posterior patterning, and Xtbx6 mediates posterior development through Wnt and FGF signaling regulation [34]. To evaluate *gpx3*’s role in posterior development signaling pathway regulation, we microinjected *Xenopus* embryos with *gpx3* MO and assessed Wnt gene expression via RT-PCR. We assessed *wnt3a*, *wnt5a*, *wnt5b*, *notch1*, *fgf8*, and *Xtbx6* expression in the excised posterior part of morphant embryos. The expressions of *wnt3a*, *wnt5b*, *notch1*, and *fgf8* were significantly reduced in *gpx3*-depleted embryos compared to control embryos (Figure 2A,B). Overall, *gpx3* is involved in posterior development by influencing Wnt, Notch, and BMP signaling pathways to regulate tailbud development.

### 3.4. Gpx3 Knockdown Does Not Perturb Early Tailbud Gene Expression 

Tailbud outgrowth is regulated by the expression of two gene sets, which are categorized as early and late genes [35,36]. Early genes (*X-delta-1*, *Xlim1*, *Xbra*, *Xnot2*, and *Xcad3*) are expressed in the future tailbud regions, while late genes (lunatic fringe, *LFNG*) are expressed at the time of tailbud extension, forming the axial tail tissues [37].

Because *gpx3* knockdown disrupted normal tail development in *Xenopus* embryos, we speculated that *gpx3* may be involved in the early tailbud gene regulation. To investigate *gpx3*’s functional role in tailbud development, we performed a loss of function experiment using the *gpx3* MO and analyzed its effect on the expression of two tailbud early genes, *Xbra* and *Xnot2*. The T-box factor, *Xbra*, is expressed in the chordoneural hinge and the posterior wall, whereas the homeobox factor, *Xnot2*, is expressed in the ventral neural tube and the chordoneural hinge. Embryos at the 4-cell stage were ventrally injected with a *gpx3* MO, and WISH was used to evaluate *Xbra* and *Xnot2* tailbud expression. Surprisingly, the WISH analysis showed that *gpx3* knockdown did not affect *Xbra* and *Xnot2* tailbud expression. The expression of both tailbud markers was the same in control embryos and *gpx3* morphant embryos, and no significant physical alterations were observed (Figure 2C). To confirm the WISH data, we performed RT-PCR using primers specific for *Xbra* and *Xnot2*. The RT-PCR results demonstrated that there were no appreciable expression differences for *Xbra* and *Xnot2* in *gpx3* MO-injected embryos (Figure 2D), consistent with WISH data. Taken together, these findings suggest that *gpx3* depletion had no functional significance in regulating early tailbud gene expression.

### 3.5. Gpx3 Regulates ROS Level in the Tailbud Region

**Figure 3:**
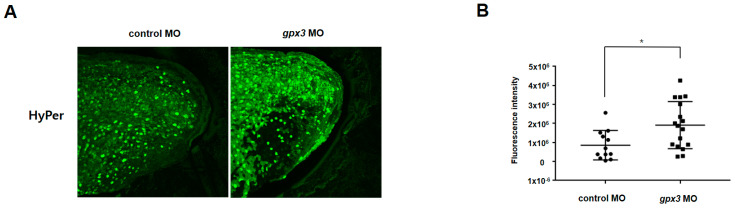
Perturbed *gpx3* leads to increased reactive oxygen species (ROS) levels. Embryos were injected with 10 ng of HyPer mRNA at the 1-cell stage. MOs were injected into the ventral regions at the 4-cell stage. (**A**) *gpx3* MO-injected embryos show increased HyPer fluorescent compared to the control. (**B**) HyPer fluorescence intensity quantification using ImageJ. * *p* < 0.05.

To assess *gpx3*’s antioxidant function, we microinjected HyPer mRNA into 1-cell staged embryos and subsequently microinjected *gpx3* MO into the embryos once at the 4-cell stage. Compared to control embryos, *gpx3* knockdown embryos showed increased HyPer fluorescent intensity, representing increased H_2_O_2_ levels related to ROS (Figure 3A,B). These results demonstrate that *gpx3* has an antioxidant function, and *gpx3* disruption in the tailbud region leads to ROS accumulation with post-anal tail defects.

### 3.6. Gpx3 Is Required for Regulating Programmed Cell Death during Xenopus Embryogenesis

**Figure 4:**
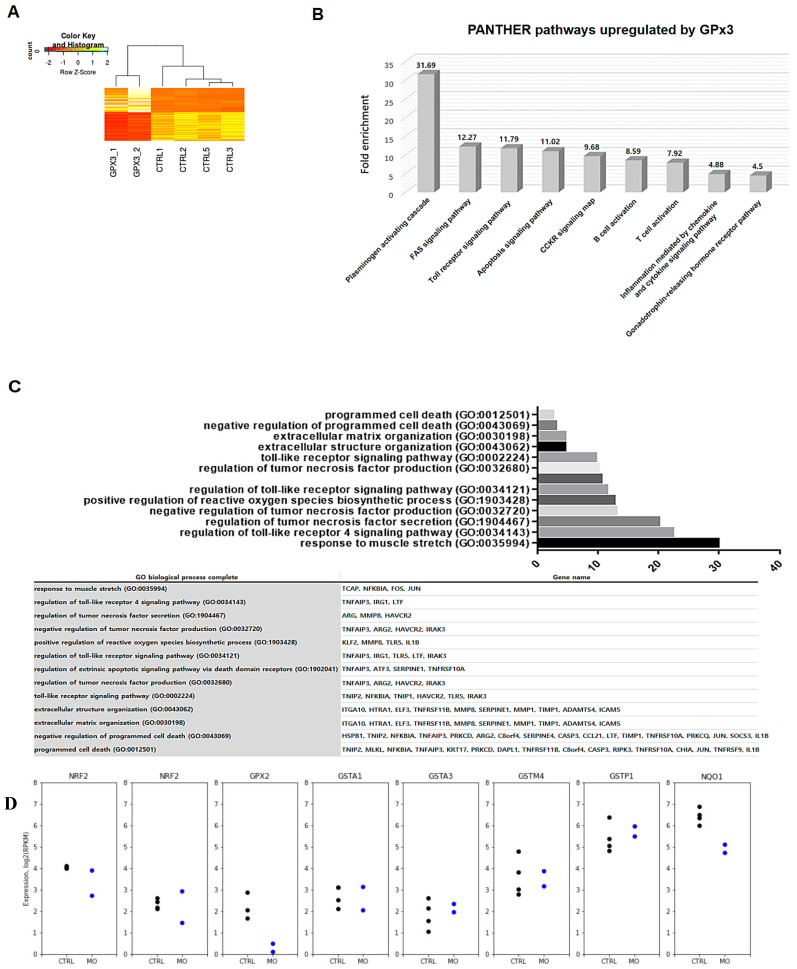
Effects of *gpx3* knockdown on the *Xenopus* embryo transcriptome. (**A**) Heat map showing significant differences between control and *gpx3* MO-injected embryos. (**B**) Transcriptome enrichment analysis using PANTHER indicated that cell death processes are significantly upregulated in *gpx3*-depleted embryos. (**C**) Gene ontology (GO-terms) and genes also indicated that genes associated with apoptosis and cell-death-related pathways were significantly affected by *gpx3* depletion. (**D**) The expression of Nrf2 and its putative targets were not affected by *gpx3* knockdown.

We performed transcriptome analysis of *gpx3*-depleted embryos to identify specific genes that are regulated by *gpx3* during *Xenopus* embryonic development. Total RNA from *gpx3* morphants was extracted and processed for transcriptome analysis. Our transcriptome analysis revealed that there were significant differences in the overall transcript expression between the control and the *gpx3* MO-injected embryos (Figure 4A). *gpx3* knockdown led to upregulation in genes related to apoptosis signaling, toll receptor signaling, and Fas signaling pathways (Figure 4B,C). Thus, our transcriptome analysis suggests that the shortened tail in *gpx3* morphants is the result of cell death in the posterior regions.

Since the knockdown of *gpx3* is associated with increased level of ROS in morphant embryos, and enhanced ROS levels can lead to activation of Nrf2. Thus, we checked our RNA-seq data to see whether *Nrf2* (*Nfe2l2*) and their putative targets were induced by *gpx3* knockdown. However, expression levels of two duplicated genes (homologs) of *nfe2l2* (*nfe2l2*.L and *nfe2l2*.S) were not changed significantly (Figure 4D). Further, we also checked the target of Nrf2 related to ROS, and they also did not show any change by depleting *gpx3* (Figure 4D). Although Nrf2 regulates many ROS detoxifying genes, our transcriptomic data showed that the *gpx3* knockdown might not activate Nrf2 regulatory network.

**Figure 5:**
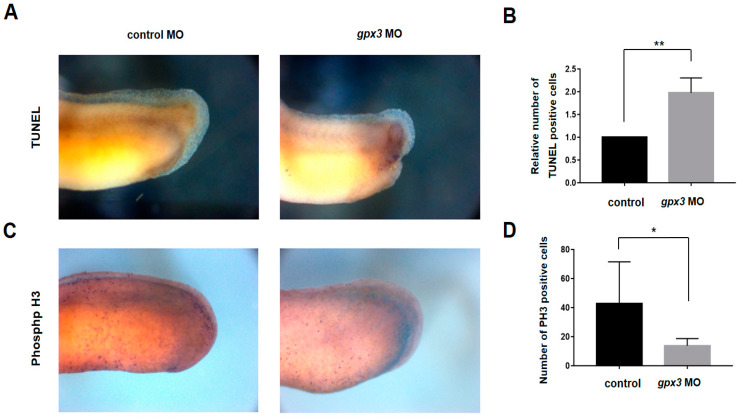
*gpx3* suppression activates apoptosis and inhibits cell proliferation in *gpx3* morphants. (**A**) The expression and number of terminal deoxynucleotidyl transferase dUTP nick end labeling (TUNEL)-positive cells were higher in *gpx3* knockdown embryos compared to control embryos. (**B**) Relative TUNEL expression, indicating a significant increase in apoptotic cells in *gpx3* morphants. ** *p* < 0.001. (**C**) *gpx3* loss resulted in reduced phospho-histone 3 (PH3)-positive cells in the tail region of *gpx3* knockdown embryos. PH3-positive cells are indicated by the color purple, whereas β-gal staining is indicated by the color blue. (**D**) The relative PH3 expression showed cell proliferation was significantly reduced by *gpx3* knockdown. * *p* < 0.05.

To verify *gpx3*’s role in cell death gene expression, a TUNEL assay and phospho-H3 staining were performed. In the *gpx3* knockdown embryos, TUNEL-positive cells were significantly increased in the tailbud regions compared to control embryos (Figure 5A,B). In contrast, cell proliferation in the tailbud region was significantly decreased in *gpx3* morphant embryos (Figure 5C,D). Together, our data suggest that *gpx3* regulates tailbud development by regulating cell death and proliferation.

## 4. Discussion

GPx3 belongs to an important class of selenoproteins [8] and is among the strongest antioxidant enzymes by catalyzing peroxide reduction and free radicals through reduced glutathione to oxidized glutathione [17]. GPx3 is well known for its roles in tumor suppression, and increased GPx3 expression may also play a protective role in cardiomyocytes by ROS detoxification [15,17,22,38]. Studies have shown that reduced GPx3 expression is associated with liver and heart disease in humans [24,39]. However, GPx3’s role in embryonic development and ontogenesis has not yet been elucidated.

In this study, we systematically assessed *gpx3*’s spatiotemporal expression pattern during embryonic development in *Xenopus*. RT-PCR analysis revealed that *gpx3* is expressed at the late blastula stage, with the highest expression at the early tailbud stage (Figure 1A). The spatial expression pattern showed that *gpx3* is localized at the tail region during *Xenopus* embryogenesis (Figure 1B). *gpx3*’s involvement in tailbud development was verified by *gpx3* MO microinjection into the embryo ventral blastomere at the 4-cell stage. *gpx3* depletion led to a shortened post-anal tail compared to control embryos (Figure 1C,D). To our surprise, *gpx3* depletion did not affect early tailbud gene expression required for tailbud development (Figure 2C,D). Instead, *gpx3* ablation perturbed gene expression associated with signaling pathways involved in tailbud outgrowth during *Xenopus* embryonic development (Figure 2A,B). These data suggest that *gpx3* is associated with signaling pathways that are required for proper embryogenesis.

Cell death and renewal are indispensable processes in multicellular organisms and are required for proper tissue and organ development [40]. Apoptosis occurs in multicellular organisms and is required for removing abnormal cells and aids in internal environment stability and multiple system development [41]. GPx3 induces apoptosis in cardiomyocytes, and excessive ROS levels can lead to apoptotic induction through caspase-3 activation [38,41]. In this study, we found that *gpx3* suppression results in increased ROS levels (Figure 3) and gene expression associated with apoptosis signaling, Fas signaling, and toll receptor signaling. These findings suggest that *gpx3* regulates embryonic development by controlling ROS levels and cell death processes (Figure 4). To further verify these findings, we performed TUNEL and PH3 staining to detect the *gpx3* effect on cell death and cell proliferation. The cell death rate was significantly increased, and cell proliferation was decreased in *gpx3* knockdown embryos (Figure 5).

## 5. Conclusions

In conclusion, apoptosis and cell proliferation are fundamental physiological processes for embryonic development. *gpx3* knockdown perturbs proper tailbud development by activating cell death and inhibiting cell proliferation. However, further work is needed to determine how *gpx3* regulates Wnt, BMP, and Notch signaling pathways. Additionally, further investigation is required to identify the regulatory mechanisms involved in *gpx3*-associated cell death during vertebrate embryonic development, as our data confirmed that *gpx3* knockdown is not associated with activation of Nrf2 regulatory network.

## Figures and Tables

**Table 1:** List of Primers for RT-PCR

Gene	Forward Primer Sequence	Reverse Primer Sequence
*odc*	5′-CAGCTAGCTGTGGTGTGG-3′	5′-CAACATGGAAACTCACACC-3′
*wnt5b*	5′-AAGCAGAGAGGCGGCATTT-3′	5′-CCGAGACACCATGGCACTT-3′
*notch1*	5′-ACCTACAAATGCTCCTGCCC-3′	5′-AACAAGGGTGGGAAGCACAA-3′
*tbx6*	5′-TATCCGGGGGAAGAGGAAGG-3′	5′-CCCTTGACTGTGGCTGATGT-3′
*Xnot2*	5′-TAATCTCCTGCACCCCCAGA-3′	5′-TTGATGCGTCGGTTCTGGAA-3′
*fgf8*	5′-CCAACTGGCAACTGAGCAAC-3′	5′-ACCGTGTCCTACCGAGAACT-3′
*Xbra*	5′-CGTGCAGTACCGGGTAGATC-3′	5′-TGGCAAATGGGTTGTGCTTG-3′
*wnt3a*	5′-TCCTCTGTGGGCTACACCAA-3′	5′-CGCCAATCACCCTGAAGTCT-3′
*wnt5a*	5′-GGCAGTGCAATGGTCTCTCA-3′	5′-GCGACATCAGCCAAGGTACT-3′
